# Bunions and Their Treatment

**Published:** 1897-02-27

**Authors:** A. H. Tubby

**Affiliations:** Assistant Surgeon to, and in charge of, the Orthopædic Department, Westminster Hospital; Surgeon to out-Patients, Evelina Hospital for Sick Children; Surgeon to the National Orthopædic Hospital.


### BUNIONS AND THEIR TREATMENT.

By A. H. Tubby, M.S.Lond., F.R.C.S.Eng., Assistant Surgeon to, and in charge of, the Orthopædic Department, Westminster Hospital; Surgeon to Out-patients, Evelina Hospital for Sick Children; Surgeon to the National Orthopædic Hospital.

This affection is known also by the synonyms of hallux extrorsus, or hallux valgus. A bunion is largely due to the use of improper boots, not necessarily of tight ones, but of those which are pointed and often too short. In some cases, however, it may be traced to osteo-arthritis and gout. These diseases, however, really give a faulty direction to the great toe, which is accentuated by narrow-pointed boots.

The term bunion should be properly applied only to the false bursa which forms over the prominent head of the metatarsal bone and the base of the displaced first phalanx. The bony enlargement is especially noticeable in osteo-arthritis, and I have on several occasions felt grating in the first metatarso-phalangeal joint.

The anatomy of the affection is as follows: The great toe is displaced outwards, so that part of the head of the metatarsal bone is uncovered; a subluxation, therefore, occurs, with the result that the internal lateral ligament is stretched, and in some cases is thickened, but is more often thinned. The external ligament of the joint is shorter and thicker than natural, and those muscle, the tendons of which are inserted into the inner side of the base of the first phalanx are stretched, and those which are inserted into the outer side are contracted. On the dorsum, the tendon of the extensor proprius pollicis is displaced externally, and by its new position takes an important part in maintaining and increasing the deformity. A recognition of this fact is important in the question of treatment. The skin on the inner side beneath the ball of the great toe is in early cases reddened, and later becomes thickened. Beneath it, in the subcutaneous tissue, a false bursa or bunion forms; the bursa is very liable to inflammation and suppuration, and the latter is sometimes followed by cellulitis. Occasionally pus may make its way into the joint and disorganisation occurs. Not unusually large and painful corns are situated on the bunion. In one patient, from whose foot I dissected out a large painful bunion, with a corn on top of it, I found on subsequent section of the part removed that, the corn extended through the whole of thickness of the bunion, that is, nearly one inch. Where the head of the metatarsal bone is not covered by the base of the phalanx, the cartilage is thin or has entirely disappeared, and much osteophytic enlargement of the head of the metatarsal bone is present, while the lipping of the base of the proximal phalanx can be frequently felt through the skin.

The symptoms are sufficiently plain. In most cases the first thing the patient complains of is the pain arising from the inflamed bursa, and it is by this symptom that the surgeon's attention is called to the part which is seen to be inflamed and prominent. In old-standing cases the altered direction of the great toe, the presence of a fluctuating swelling, and of corns over the inner side of the head of the metatarsal bone suffice for the diagnosis. Occasionally the affection is mistaken for gout, but a little care will distinguish an inflamed bunion from an acute attack of podagra. As the great toe is being displaced it rides over the second toe, and depresses the second and third phalanx, so that hammer-toe complicates the deformity. In cases of hallux valgus or extrorsus flat foot is often present. It is to be noted that women suffer more frequently than men, three times as often, and both feet are simultaneously affected, though not to the same degree.

**Treatment.**—Prophylactic. Pointed boots must be absolutely forbidden. It is not of much avail to discuss with the patient whether the boots he or she is wearing are pointed or not. An outline of the sole of the boot to be worn must be given, and directions that the patient should go to a rational bootmaker who will carry out instructions. The sole of the boot should be as broad as the sole of the foot when it is placed on the ground, and when the weight of the body is being borne on it. If any displacement exist, the inner side of the upper leather should be so blocked out as to give ample room to the great toe and to the prominence of the metatarso-phalangeal joint.

**Curative Treatment.**—This, in slight cases, consists in wearing boots made on the lines just suggested, together with the application of cold and soothing lotions to the inflamed parts, and in the use of some arrangement whereby the great toe is kept away from the second toe. In early stages nothing answers so well as a divided or digitated sock with a separate stall for the great toe. Some advise the use of a "post" between the first and the second toes, and this has been found in some cases to answer well, but a little difficulty will occur in bringing the great toe into the proper place for it. Steadily pulling the great toe inwards night and morning, when persevered in, soon brings it into better position. An excellent arrangement is made for these cases, namely, a leather cap or thimble fitting over the great toe, secured by a tape which passes along the inner border of the foot and around the heel by an elastic insertion, which is fastened at the outer border of the foot. A somewhat more elaborate apparatus consists of a metal plate adjusted to the foot at the instep. At the lower part of the plate a spring is jointed and curved convexly towards the toe, having a division in the spring over the bunion to avoid pressure. When the patient is unable to afford a special apparatus, a wedge-shaped pad of lint fixed between the toes is of service, and the first toe may be further separated from the second by drawing it away by strapping secured round it, and passing back towards the heel; or a splint of rubber or pasteboard may be fixed to the inner margin of the foot and toe, thus pulling the latter inwards.

When the deformity is severe, the bursa large and painful, and subject to recurring attacks of inflammation, operative procedures are necessary. In many cases, before the toe can be brought into proper position, it is necessary to divide the tendon of the extensor proprius pollicis, but, in fact, this forms a part of the succeeding methods.

When the cases are moderately severe, I divide the extensor proprius pollicis tendon, and then make a linear incision along the inner side of the bunion and in a line with the long axis of the foot. I then dissect out the large and inflamed bursa and open the joint. With a chisel I remove the prominences of the bone on the inner side of and below the head of the metatarsal bone and the base of the first phalanx. In severer cases, in place of chiselling off the bony prominences, I remove the head of the first metatarsal bone, leaving the phalanx intact. But in the severest cases I remove both the head of the metatarsal bone and the base of the first phalanx. In three instances I have taken away the bony prominences, in five instances I have removed the head of the metatarsal bone, and in four instances I have excised the base of the first phalanx as well as the head of the first metatarsal bone, in all cases with success, both as to the relief of pain and improvement in locomotion. The important point about these operations is, of course, the question of proper movement of the great toe afterwards, and in no instance has there been any difficulty on this point. It seems, then, that where bunions are very painful and prone to suppuration, and give rise to great difficulty in locomotion, these small surgical procedures are fully justified on account of the immediate and permanent relief they afford.

